# Implementation of the Swedish *Guideline for Prevention of Mental ill-health at the Workplace*: study protocol of a cluster randomized controlled trial, using multifaceted implementation strategies in schools

**DOI:** 10.1186/s12889-019-7976-6

**Published:** 2019-12-11

**Authors:** Lydia Kwak, Caroline Lornudd, Christina Björklund, Gunnar Bergström, Lotta Nybergh, Liselotte Schäfer Elinder, Kjerstin Stigmar, Charlotte Wåhlin, Irene Jensen

**Affiliations:** 1 Unit of Intervention and Implementation Research for worker health, Institute for Environmental, MedicineKarolinska Institutet, Stockholm, Sweden; 2 Department of Learning, Informatics, Management and Ethics, Medical Management Centre, Karolinska Institutet, Stockholm, Sweden; 3 Department of Occupational and Public Health Sciences, Faculty of Health and Occupational Studies, Centre for Musculoskeletal Research, University of Gävle, Gävle, Sweden; 4 Department of Global Public Health, Karolinska Institutet, Stockholm, Sweden; 5 Centre for Epidemiology and Community Medicine, Stockholm, Sweden; 6 Department of Health Sciences, Lund University, Lund, Sweden; 7 Skåne University Hospital, Lund, Sweden; 8 Occupational and Environmental Medicine Center, and Department of Clinical and Experimental Medicine, Linköping University, Linköping, Sweden

**Keywords:** Implementation strategies, Teachers, Mental ill health, Stress, Guideline, Adherence

## Abstract

**Background:**

Given today’s high prevalence of common mental disorders and related sick leave among teachers, an urgent need exists for a more systematic approach to the management of social and organizational risk factors within schools. In 2015, we launched the first Swedish occupational health guideline to support a structured prevention of these risks at the workplace. The existence of guidelines does however not guarantee their usage, as studies show that guidelines are often underused. Knowledge is therefore needed on effective implementation strategies that can facilitate the translation of guidelines into practice. The primary aim of the randomized waiting list-controlled trial described in this study protocol is to compare the effectiveness of a multifaceted implementation strategy versus a single implementation strategy for implementing the *Guideline for the prevention of mental ill-health at the workplace* within schools. The effectiveness will be compared regarding the extent to which the recommendations are implemented (implementation effectiveness) and with regard to social and organisational risk factors for mental ill-health, absenteeism and presenteeism (intervention effectiveness).

**Methods:**

The trial is conducted among primary schools of two municipalities in Sweden. The single implementation strategy is an educational strategy (an educational meeting). The multifaceted strategy consists of the educational meeting, an implementation team and a series of workshops. The outcome measure of implementation effectiveness is guideline adherence. The primary outcome of intervention effectiveness is exhaustion. Secondary outcomes include demands at work, work organization and job contents, interpersonal relations and leadership, presenteeism, work performance, recovery, work-life balance, work-engagement, self-reported stress, self-perceived health, sickness absence and psychosocial safety climate. Process outcomes as well as barriers and facilitators influencing the implementation process are assessed. Data will be collected at baseline, 6, 12, 18 and 24 months by mixed methods (i.e. survey, focus-group interviews, observation).

**Discussion:**

The study described in this protocol will provide valuable knowledge on the effectiveness of implementation strategies for implementing a guideline for the prevention of common mental disorders within schools. We hypothesize that successful implementation will result in reductions in school personnel’s perceived social and organizational risk factors, mental ill-health and sick-leave.

**Trial registration:**

ClinicalTrials.gov ID: NCT03322839 (trial registration: 09/19/2017).

## Background

### Background and rationale

Globally, common mental disorders (CMDs), such as depression, anxiety and adjustment disorders, are highly prevalent [1]. The Organization for Economic Cooperation and Development (OECD) has estimated that approximately 20% of the working-age population is living with a CMD at any given time [2]. The consequences of the high prevalence of CMDs for employers are enormous, including high rates of sickness absence, significant losses in productivity at work and individual suffering [2]. A professional group that is especially vulnerable for CMDs are schoolteachers [3–5]. Teachers have been identified as being the professional group with the highest risk of poor mental health compared to other professions [6]. Evidence shows that, in addition to personal factors, social and organizational risk factors at the workplace, such as high demands at work, low job-control and low support from colleagues and superiors increase the risk for CMDs [7, 8]. The systematic management of social and organizational risk factors at the workplace is therefore commonly recommended as a strategy to prevent CMDs [9, 10]. The social and organizational work environment of teachers is characterized by high workload/work intensity, role overload, increased class size per teacher, unacceptable pupils’ behavior, bad school management and/or lack of support from management, resulting in a high risk for mental ill-health and consequently sick leave [11].

Few workplaces to date, however, systematically modify or eliminate sources of social or organizational risks inherent in the work environment. When it comes to schools, a recent report by the Swedish Work Environment Authority showed that most Swedish schools had severe shortages in their social and organizational risk management, including a lack of assessment of unhealthy work demands [12]. Rather, the focus is often primarily on remedial measures for employees affected by CMDs [13, 14]. Even though evidence suggests favorable effects at both individual and organizational level of implementing organizational level interventions aimed at addressing the problems at source [15]. In order to support workplaces with the management of their social and organizational work environment and prevent CMDs, we launched the first Swedish occupational health guideline to support the prevention and treatment of work-related mental ill-health at the workplace [16]. The guideline is based on the best available evidence [e.g. [17–19]] and has been compiled in a cross-disciplinary collaboration between employers, occupational health service practitioners and researchers.

The mere existence of guidelines does, however, not guarantee their usage [20]. Efforts to disseminate guidelines via e.g. websites, publication of consensus statements, or mass mailings often result in relatively low adoption, resulting in only small changes in practice. There is therefore a clear need for well-designed studies that aim to facilitate the implementation of guidelines into practice, including occupational health guidelines aimed at the prevention of CMDs. Various models demonstrate that multiple contextual factors, so-called barriers and facilitators, affect how efficiently guidelines and recommendations are implemented. These factors can be related to characteristics of the guidelines themselves (e.g. complexity), characteristics of the users (e.g. knowledge), the internal environment (e.g. the organization’s capacity for change) and the external environment (e.g. existence of national policies) [21]. In 2012, Eurofond and the European Agency for Safety and Health at Work (EU-OSHA) undertook a European assessment of barriers and facilitators for managing social and organizational risks at the workplace [22, 23]. According to their findings and supported by others [24–26], factors that can hinder the management of these risks were primarily related to characteristics of the users (insufficient education and knowledge) and to the internal environment (lack of technical support and guidance, as well as lack of resources) [22–26].

In order to facilitate guideline implementation and adherence to recommendations, implementation strategies are required that target identified barriers and facilitators [27]. To date, there is no consensus on which implementation strategies are most effective in facilitating guideline implementation. It is, however, recognized that active strategies are needed to improve guideline adherence [28]. Research has also shown that multifaceted strategies appear to be more effective than single strategies [29], because multiple implementation barriers are targeted at the same time. However, previous studies examining the effectiveness of multifaceted strategies are inconclusive [28, 30, 31]. One reason for this is that the multifaceted strategies evaluated often were aimed at only one barrier (e.g. lack of knowledge) and therefore not successful in changing behavior [28]. Behavior change is more likely to occur if implementation strategies are targeting different types of barriers simultaneously (e.g. lack of knowledge and lack of organizational support). Another limitation of previous studies evaluating implementation strategies’ effectiveness is their lack of a clear theory-base [32]. A recent review on the use of theory (or models and frameworks) to plan or evaluate guideline implementation concluded that only half of the guideline implementation studies were based on theory, models or conceptual frameworks and only few provided details about how they were used [33]. In this study, we employ Michie and colleagues’ COM-B model to inform the design of implementation strategies [34]. COM-B is used to identify which components of the behavior system (motivation, capability or opportunity) need to be changed to achieve a behavior change [34]. The COM-B model posits that behavior is a function of three components: Capability (C), Opportunity (O), and Motivation (M). Capability refers to the ability to engage in the cognitive or physical processes necessary for the behavior, e.g. knowledge and skills. Motivation refers to those brain processes that direct behavior, and include reflective and automatic motivation, e.g. analytical decision-making and emotional responses. Opportunity refers to those factors that lie outside the individual and influence behavior, e.g. social support and prompts [34]. Implementation strategies are chosen based on Powell and colleagues’ refined compilation of implementation strategies. The compilation has been recommended as a guide to facilitate the development of multifaceted, multilevel implementation strategies that are tailored to local contexts [27]. To evaluate guideline implementation, we use Proctor and colleagues’ conceptual framework for dissemination and implementation [35]. Proctor’s framework defines evidence-based intervention strategies (e.g. guidelines) and separate strategies for implementing those intervention strategies into practice. Moreover, the framework defines different levels of change that an intervention is addressing: the larger system or environment, the organization, a group or team, or the individual. Finally, we will use the Consolidated Framework for Implementation Research (CFIR) [21] to identify barriers and facilitators that may influence the implementation process. CFIR is a meta-theoretical framework that consists of five domains (the intervention, inner setting, outer setting, the individuals involved and the implementation process) that can be used to identify barriers and facilitators [21].

**Fig. 1:**
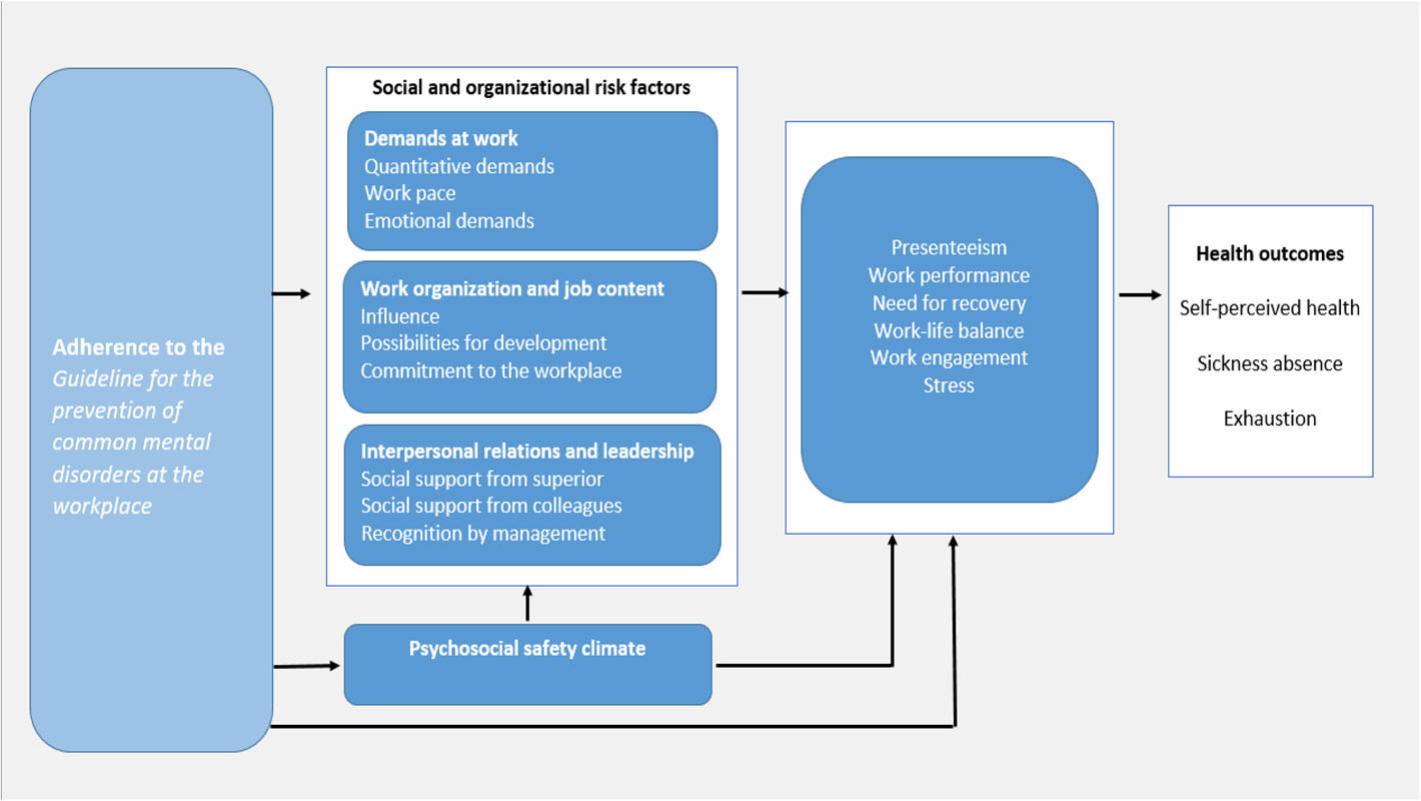
Programme theory based on Schelvis et al., 2013 [36]

A randomized controlled trial with two arms will be conducted comparing an implementation intervention with a single strategy versus one with multifaceted strategies (educational strategy, implementation teams and workshops). Information will be collected on both implementation effectiveness, and intervention (guideline) effectiveness (i.e. reduction of risk factors for mental ill-health). This will provide valuable knowledge on whether adherence to the recommendations in the guideline will result in reductions in employees’ perceived social and organizational risk factors, mental ill-health and sick-leave. The programme theory is depicted in Fig. 1. The trial will be conducted within schools with the aim to prevent mental ill-health and sick leave among teachers. Globally, teachers have a high prevalence of CMDs and related sick-leave [37–39].

### Objectives

The primary objective of this study is to compare the effectiveness of a multifaceted implementation strategy versus a single implementation strategy for implementing the *Guideline for the prevention of mental ill-health at the workplace* within schools. The effectiveness will be compared with regard to the extent to which the recommendations are implemented within the schools (implementation effectiveness) and with regard to social and organisational risk factors for mental ill-health, absenteeism and presenteeism (intervention effectiveness).

The hypothesis is that schools that receive support in implementing the guideline through a multifaceted implementation strategy are more responsive to working in a structured and systematic manner with the management of social and organisational risks at their workplace and consequently reduce risk factors for mental ill-health, compared to schools that receive support through a single implementation strategy based on education.

The secondary objective is to evaluate the implementation process of the guideline within schools. This will be done by collecting qualitative and quantitative data on implementation outcomes as defined by Proctor and colleagues [35]. Moreover, contextual factors (barriers and facilitating factors) that may influence the implementation process will be identified. This is done by collecting qualitative and quantitative data on barriers and facilitators in accordance with the CFIR [21]. This will give a better understanding of factors that may influence the process and will inform future necessary adaptation of the implementation strategies.

### Trial design

**Fig. 2:**
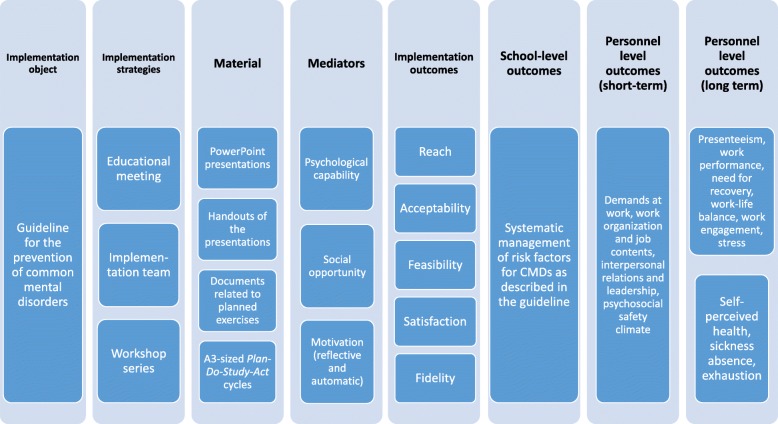
Program theory for the implementation intervention including mediators and outcomes

The study is a 12-month cluster-randomized waiting list-controlled trial with before and after measurements involving 20 primary schools in two municipalities in Sweden. Randomization (1:1 allocation to intervention and waiting list control) is conducted at the school level. Primary schools are randomized by a computer-generated randomization-list to either the ARM 1 or ARM 2 after stratification in blocks by municipality and school size (number of students per school). Schools in ARM 1 will receive all three implementation strategies during the first year of the study period while schools in ARM 2 will receive only the educational strategy in year 1 and continue with the other strategies during year 2 (Fig. 2). The implementation strategies target the managerial level of first line managers i.e. school-principals. All employees of the participating schools will be invited to complete outcome-questionnaires at baseline and 6-, 12-, 18- and 24-months follow-up during working hours.

### Trial registration

This trial has been registered in ClinicalTrials.gov (NCT03322839, date assigned: August 2017). Moreover, this study has been approved by the Ethical Committee of Stockholm (2017/984–31/5).

## Methods

### Schools, participants, interventions, and outcomes.

#### Study setting

The study is being conducted among public primary schools of two municipalities in Sweden. One of the municipalities is located in the greater Stockholm area and has seven public primary schools with a number of pupils ranging from 200 to 800. The other municipality is located 84 km from Stockholm city centre and has 13 public primary schools with a number of pupils ranging from 15 to 500. The schools give a good representation of different geographical, socioeconomic as well as urban and rural areas. A list of study sites can be obtained from the corresponding author, after agreement with the participating municipalities.

### Schools, participant recruitment and inclusion and exclusion criteria.

#### Recruitment of schools

In the first step, schools were recruited through advertisements in newsletters, such as the newsletter of The Swedish Association of School Principals and Directors of Education and the newsletter of the Swedish Union of Teachers. In addition, we advertised via social media such as Twitter, Facebook and our work-environment blog and university webpage. In the second step, an oral presentation describing the study was given to those municipalities and their school principals that expressed interest in the study.

#### School eligibility criteria

Only public primary schools are included.

#### Recruitment of participants

Several strategies are used to achieve adequate participant enrolment to reach the target sample size. Firstly, the research-team visits each participating school and presents the project. The scheduling of the oral presentation (e.g. before school-start) is done by the school-principals, to ensure that all school personnel can be present. Secondly, the research-team sends a link to a videoed version of the presentation by email to all personnel of the participating schools, which allows reaching those individuals who are unable to be present during the presentation. During the visit to the schools, all school personnel are given an information letter describing the purpose of the study, research approach, voluntary participation and data collection process. The information letter also explains that participants can withdraw from participation at any time of the study. Moreover, school personnel are given the opportunity to ask questions to the research team if anything is unclear. Those individuals who decide to participate in the study are accordingly asked to complete an informed consent form, the completed form is then returned to the research-team.

#### Participant eligibility criteria

Eligibility criteria for individual participants include that only individuals employed by the participating schools are eligible to participate; this includes teachers, but also administrative personnel. Individuals who work at the schools, but who are not employed by the school (e.g. cleaning and kitchen personnel) are excluded, as they do not fall under the management of the school-principal.

### Interventions

#### Implementation object

The implementation object that will be implemented is the *Guideline for the prevention of mental ill-health at the workplace* [16]*.* The guideline represents best practice in the field of the prevention of CMDs at the workplace [e.g. [17–19]] and has been compiled in a cross-disciplinary collaboration between employers, occupational health service practitioners and researchers. The guideline complies with the Swedish Work Environment Authority’s organizational and social work environment provisions (AFS 2015:4). The guideline contains two parts. The first part aims to prevent work-related mental ill-health from an organizational perspective and is suitable for supporting employers with their preventive work. The second part focuses on the treatment of mental ill-health and is solely suitable for the occupational health services. This study will focus on the implementation of the first part of the guideline. Table 1 summarizes the recommendations specified in the guideline and corresponding target behaviors. The target behaviors are specified by the research team and represent those behaviors that are likely to bring about change.

**Table 1:** Overview of the recommendations of the *Guideline for the prevention of mental ill-health at the workplace* and corresponding target-behaviors

Recommendation	Target-behaviors
1.Workplaces have well-established policies related to the social and organizational risk management	Develop new policies related to the social and organizational risk management
	Revise existing policies related to the social and organizational risk management
	Disseminate policies related to the social and organizational risk management
	Apply policies related to the social and organizational risk management
2.Employers have knowledge on the relationship between social and organizational risks and mental ill-health	Formulate the knowledge employers need to have regarding the relationship between social and organizational risks and mental ill-health
	Identify appropriate courses that can supply employers with knowledge on the relationship between social and organizational risks and mental ill-health
	Apply for courses that can supply employers with knowledge on the relationship between social and organizational risks and mental ill-health
3.Workplaces regularly assess their social and organizational work environment and intervene on identified social and organizational risk factors	Plan the assessment of the social and organizational work environment
	Assess the social and organizational work environment
	Present the results of the assessment of the social and organizational work environment to all employees
	Identity priority areas in group-discussions based on the results that need to be intervened upon
	Develop an action-plan describing activities aimed at the priority areas
	Execute the intended activities
	Continuously follow-up the action-plan

#### Development of the implementation strategies

In this study, a multifaceted implementation strategy (ARM 1) is compared with a single implementation strategy (ARM 2). The single implementation strategy consists of a one-day educational meeting. The multifaceted strategy consists of the educational meeting, establishment of an implementation team and a series of workshops. The research team develops the implementation strategies following a systematic and theory-based approach, consistent with French and colleagues´ approach for developing theory-informed implementation strategies [40]. In the first step, target behaviors to change are specified as described in Table 1. In the second step, Michie and colleague’s COM-B model [34], existing literature on barriers to the management of social and organizational risk at the workplace (e.g. [23]) and two planning workshops are used to identify factors to target. Existing taxonomies of implementation strategies [27] are used to select appropriate implementation strategies. Two representatives of the research-team hold the planning workshops (2.5 h each) prior to the start of the project (Maj – June 2017) with school principals and the school’s health and safety officers. The aim of the planning workshops is to tailor the implementation strategies to the needs of the participating schools. Information is gathered on 1) behaviors, attitudes and knowledge needed to be able to adhere to the recommendations of the guideline and 2) barriers and enablers that can influence adherence to the recommendations. Moreover, to ensure feasibility of the implementation strategies, practicalities are discussed regarding the duration of the educational meeting and workshops, as well as who should participate in the educational meeting and workshop series. Table 2 gives an overview of identified barriers and enablers to the implementation of the guideline. The COM-B model is used to further guide the development of the content of implementation strategies [34]. The implementation strategies target the different COM-B constructs, as described in Table 3.

**Table 2:** Barriers and facilitators identified during the planning workshops and in the literature

Barriers	Enablers
Capability	Capability
Lack of knowledge on how to manage social and organizational risks at the workplace	Knowledge on the guideline recommendations
Motivation	Motivation
Difficult to prioritize	Motivated, engaged, enthusiastic
Lack of time	Have the right mind-set
Difficult to execute plans	Systematic approach, structure
Unclear professional role	
Opportunity	Opportunity
Lack of support	Social support from colleagues and organization

**Table 3:** Implementation strategies and relation to COM-B constructs and content.

Implementation strategies	COM-B	Content
Educational meeting	Motivation	Introduction by the research-team, including the aim and structure of the meeting
	Psychological capability	Research-team presents the content of the guideline
	Reflective motivation	Exercise 1: schools discuss if there is a need to adapt the recommendations to the school-context
	Psychological capability	Exercise 2 (world-café): schools exchange knowledge regarding how they adhere to the recommendations of the guideline and possible barriers and facilitators of adherence.
	Reflective motivation	Exercise 3: each school chooses a recommendation to implement and discusses the advantage of adhering to the recommendation.
	Reflective motivation	Exercise 4: each school develops an action plan for the implementation of the chosen recommendation
	Social opportunity Motivation	Exercise 5 (devils-advocate): in pairs - schools peer review each other’s action plan.
	Psychological capability Motivation	Research-team presents the concepts of barriers and facilitators. Group discussion on what can influence the implementation of the formed action plans and on what can ensure a successful implementation.
	Automatic motivation	Research-team asks each school to answer a set of motivational coaching questions aimed creating motivation for implementing the guideline.
Implementation team	Social opportunity	The members of the implementation team provides social support within team.
	Social opportunity	The implementation team provides social support, modelling and social comparison between teams.
	Reflective motivation	The *Plan-Do-Study Act cycles* give implementation teams a structured approach to the implementation of the guideline’s recommendations.
	Social opportunity	The communication plan will give implementation teams the opportunity to specify the support they need from the municipality’s educational board to facilitate the implementation process.
Workshop 1	Reflective motivation	Introduction, each school describes which recommendation they chose to implement under the educational-meeting, and any activities that have already been undertaken.
	Psychological capability	Research-team presents recommendation 1 and specified target-behaviors
	Psychological capability	Research-team presents the concept of SMART-goals
	Reflective motivation	Exercise 1 (PLAN): implementation team writes a SMART-goal for a recommendation they are planning to implement
	Psychological capability, reflective motivation	Exercise 2 (PLAN): implementation teams conduct a planning exercise, specifying in small detailed steps how they are planning to reach their SMART-goal. The exercise results in a detailed action-plan describing the different steps, when they will be undertaken and who is responsible.
	Social opportunity	Plenary discussion on support and communication, both between the implementation teams and from the municipality’s educational board.
Workshop 2	Reflective motivation	Each implementation team briefly presents if they carried out their specified plan (DO), which barriers and facilitators influenced execution (STUDY) and what needs to be adapted in the plan (ACT).
	Psychological capability	Research-team presents the concept of behavior-change and factors that can influence behavior change.
	Reflective motivation, social opportunity	Exercise 1: in pairs - implementation teams discuss barriers that influenced execution (STUDY) and possible solutions to these barriers.
	Reflective motivation	Exercise 2: on the basis of exercise 1 each implementation team adapts their action plan (ACT) or develops a new action-plan (PLAN)
	Social opportunity	Plenary discussion with a representative of the municipality’s educational board on support needed for successful implementation.
	Reflective motivation	Each implementation team briefly presents their (updated) plan (PLAN)
Workshop 3	Psychological capability, automatic motivation	Research-team discusses the importance of having routines in place to ensure that implemented changes are sustained over time.
	Reflective motivation	Each implementation team briefly presents if they carried out their specified plan (DO), which barriers and facilitators influenced execution (STUDY) and what needs to be adapted in the plan (ACT).
	Reflective motivation	The implementation team adapts their action plan (ACT) or develops a new action-plan (PLAN)
	Reflective motivation	Exercise 1: (backward brainstorm) plenary exercise identifying barriers that hinder individuals from following routines. The exercise results in a consensus of what an optimal routine should look like.
	Reflective motivation	Exercise 2: each implementation team choses a recommendation for which they would like to develop a routine.
	Reflective motivation	Exercise 3: each implementation team develops a routine for the chosen recommendation - describing how the recommendation can become a reoccurring routine (PLAN), for example how can we ensure that the assessment of social and organizational risk at our workplace becomes a reoccurring event.
Workshop 4	Reflective motivation	Exercise 1: implementation team reflects over how they currently work with recommendation 3.
	Reflective motivation, social opportunity	Plenary discussion on current adherence to recommendation 3.
	Psychological capability, automatic motivation	Research-team presents recommendation 3 and specified target-behaviors
	Reflective motivation	Exercise 2 (PLAN): implementation team writes a SMART-goal for recommendation 3.
	Reflective motivation	Exercise 3 (PLAN): implementation team conduct a planning exercise, specifying in small detailed steps how they are planning to reach their SMART-goal. The exercise results in a detailed action-plan describing the different steps, when they will be undertaken and who is responsible.
	Reflective motivation	Each implementation team briefly presents their plan (PLAN)
Workshop 5	Reflective motivation	Each implementation team briefly presents if they carried out their specified plan (DO), which barriers and facilitators influenced execution (STUDY) and what needs to be adapted in the plan (ACT).
	Psychological capability, automatic motivation	Research-team further presents recommendation 3 and specified target-behaviors.
	Reflective motivation	Exercise 1: The implementation teams conduct an exercise related to recommendation 3. The exercise is aimed at supporting the implementation teams with prioritizing areas of their social and organizational work environment that need to be improved.
	Reflective motivation	Exercise 2: The implementation team adapts their action plan (ACT) or develops a new action-plan (PLAN) aimed at the implementation of recommendation 3.
	Reflective motivation	Exercise 3: The implementation team reflects over how they will continue with the implementation of the recommendations and adherence to the recommendations. Moreover, the implementation teams plans for the coming school-year, e.g. in what way will the implementation team continue, how often will they meet, are additional members needed etc.

### Implementation strategies

#### Educational strategy

The educational strategy is a full-day educational meeting (6.5 h) held at two occasions in October 2017, once for each of the participating municipalities. One of the meetings is held at the university and the other at the city hall. Prior to the meeting school principals receive an invitation by email describing the purpose of the meeting and the requirements for participation. The requirements include forming a group of 4–5 individuals (e.g. teacher union representatives, teacher representatives, health and safety officers, assistant school principals), discussing with the group the current approach to the management of social and organizational risk factors at their school and participating in the meeting with the group. Two members of the research team (a researcher with implementation expertise and a licensed psychologist with occupational health expertise) hold the educational meeting. At the start of the meeting, participants receive the *Guideline for the prevention of mental ill-health at the workplace* and a compendium, which includes handouts of the presentations and documents related to the planned exercises. The educational meeting includes PowerPoint presentations, material for plenary discussions and group-exercises. Table 3 provides an overview of the content of the educational meeting and the constructs of the COM-B they are targeting. The programme theory for the implementation strategies is depicted in Fig. 2.

#### Implementation team

Directly after the educational meeting in October 2017 school principals are instructed to form an implementation team among those individuals who participate in the educational meeting (3–5 individuals). The implementation team is responsible for leading the implementation of the guideline within their own school during the study period and beyond. To facilitate the implementation process, implementation teams use *Plan-Do-Study-Act* improvement cycles [41]. During *Plan* the implementation team specifies the action-plan to implement a guideline recommendation within their school (one recommendation at a time). During *Do* the implementation team carries out the action-plan as specified. During *Study* the implementation team assesses whether the action-plan was executed as planned and which factors influenced execution. During *Act* the implementation team makes changes to the plan, if needed, to further improve implementation. The implementation team repeats the *Plan-Do-Study-Act cycles* until the chosen recommendation is implemented and then continues with a new *Plan-Do-Study-Act cycle* for the next recommendation. In addition, implementation teams are responsible for establishing a communication plan specifying how they will communicate with their municipality’s educational board during the implementation process. Table 3 describes the relationship between the implementation team and the constructs of the COM-B model. Implementation teams receive support through a series of five workshops.

#### Workshop series

At each of the municipalities, the research-team holds a series of five workshops (2.5 h per workshop) for the implementation teams (October 2017–June 2018). The first workshop is given 2 weeks after the educational meeting. A researcher with implementation expertise and a licensed psychologist with occupational health expertise give the first three workshops; the same researcher with implementation expertise and a researcher with expertise in the guideline recommendations give the last two workshops. The workshops provide the implementation teams with knowledge and skills regarding 1) the recommendations of the guideline, 2) implementation processes, and 3) *Plan-Do-Study-Act cycles*. During workshop 1 the research team presents recommendation 1 and specified target-behaviors, during workshop 2 recommendation 2 and specified target-behaviors are presented, during workshop 3 the research team presents routines for how to sustain implemented changes over time, during workshop 4 and 5 recommendation 3 and specified target-behaviors are presented. With regards to the implementation process the research team presents the concept of SMART-goals (Specific, Measurable, Achievable, Relevant and Time-bound), behavior change and barriers and facilitators that can influence behavior change, and strategies needed to target the identified barriers and facilitators. Moreover, implementation teams work with their *Plan-Do-Study-Act* cycles: developing a detailed plan for implementing the recommendations (*Plan),* discussing the success of carrying out their plan (*Study*), and adapting the plan if necessary (*Act*). In between the workshops, implementation teams carry out the action-plan as specified (*Do*). Every workshop includes PowerPoint presentations, plenary discussions and group-exercises. At the start of each workshop, participants receive a compendium, which includes handouts of the presentations and documents related to the planned exercises.

Documents for workshop 1 and 2 include worksheets to form SMART-goals, A4-sized papers to write down the different steps towards reaching the goal and empty action plans to fill in. Documents for workshop 3 include written information on routines, the formulation of routines and examples of existing routines in this field, and a worksheet on which the implementation team can formulate their own routine. Documents for workshop 4 include a detailed description of recommendation 3, including an example of how to schedule a meeting at the workplace to discuss and prioritize identified social and organizational risk factors. Moreover, empty action plans are provided. Documents for workshop 5 include empty action plans. Additional documents for workshop 2–5 include A3-sized *Plan-Do-Study-Act* cycles, on which the research-team notes the implementation teams’ progresses. Table 3 provides an overview of the content of the workshops and the constructs of the COM-B they are targeting.

### Criteria for discontinuation and monitoring

It is not possible to modify the allocated implementation strategies for a given school. Individual participants can discontinue their participation during the study trial and have their results removed. Two persons from the research-team will deliver the intervention strategies to the schools, reminding each other to adhere to the protocol. Detailed schedules for the educational-meeting and for each workshop are developed to further improve adherence to the protocol. The schedule includes a description of the time line, different components (e.g. group work, presentation) and who is responsible for which components. The schedule includes a fidelity column where any deviations are noted. The research-team will be responsible for monitoring whether the implementation interventions are delivered according to the protocol. Procedures for monitoring adherence include making notes in the fidelity column when deviations occur, for example regarding time or content. Moreover, a research-log is kept describing deviations and possible reasons for deviations.

### Contamination care

To reduce the risk of contamination between schools of ARM 1 and 2 within the same municipality, schools of ARM 1 are encouraged not to discuss the project with schools of ARM 2. Every 6 months during the trial period, the research team has a telephone meeting with the school principals to collect data regarding schools’ exposure to other interventions and potential contamination for control schools.

### Outcomes

An overview of the measurement variables, method of data-collection, data-source and time-point for each measure is described in Table 4.

**Table 4:** Measurement variables, method of data-collection, data-source and time-points

Measure	Method of data collection	Data source	Time-point
Demographic data			
*1. Implementation effectiveness*			
Guideline adherence	Questionnaire (developed by research team)	School management, school personnel	T0, T1, T2, T3 and T4
*2. Intervention effectiveness*			
Demands at work -Quantitative demands -Work pace -Emotional demands	Questionnaire (COPSOQ-II [42])	School management, school personnel	T0, T1, T2, T3 and T4
Work organization and job contents -Influence -Possibilities for development -Commitment to the workplace	Questionnaire (COPSOQ-II [42])	School management, school personnel	T0, T1, T2, T3 and T4
Interpersonal relations and leadership -Social support from superior -Social support from colleagues -Recognition	Questionnaire (COPSOQ-II [42])	School management, school personnel	T0, T1, T2, T3 and T4
Presenteeism	Questionnaire [43]	School management, school personnel	T0, T1, T2, T3 and T4
Work performance	Questionnaire (WPAI:GH [44, 45])	School management, school personnel	T0, T1, T2, T3 and T4
Need for recovery	Questionnaire [46]	School management, school personnel	T0, T1, T2, T3 and T4
Work-life balance	Questionnaire (COPSOQ-II [42])	School management, school personnel	T0, T1, T2, T3 and T4
Work engagement	Questionnaire (UWES [47])	School management, school personnel	T0, T1, T2, T3 and T4
Stress	Questionnaire [48]	School management, school personnel	T0, T1, T2, T3 and T4
	Mobile-phone text message-question [49]	School management, school personnel	Monthly during 12 months
Self-perceived health	Questionnaire (SF-12 health [50])	School management, school personnel	T0, T1, T2, T3 and T4
Short-term sick leave	Questionnaire [45]	School management, school personnel	T0, T1, T2, T3 and T4
Long-term sick leave due to mental ill-health	Register-data Swedish Insurance Agency	School management, school personnel	1-year before baseline and during the study period
Exhaustion	Questionnaire (OLBI [51])	School management, school personnel	T0, T1, T2, T3 and T4
Psychosocial safety climate	Questionnaire (PSC [52])	School management, school personnel	T0, T1, T2, T3 and T4
*3. Implementation process*			
Reach	Participation list	Research team	During the educational meeting and workshops
Acceptability	Interview	Implementation team members	T2, T4
Feasibility	Interview	Implementation team members	T2, T4
Satisfaction	Questionnaire	Participants educational meeting, workshops	After the educational meeting, after each workshop
	Interview	Implementation team members	T2, T4
Fidelity	Observation, meeting notes, work documents	Research team	During educational meeting and workshops
Barriers and facilitators	Interview (CFIR [21])	Implementation team members	T2, T4

T0: Baseline, T1: 6 months after baseline, T2: 12 months after baseline, T3: 18 months after baseline and T4: 24 months after baseline.

### Timeline of the enrolment, implementation strategies and assessments

**Fig. 3:**
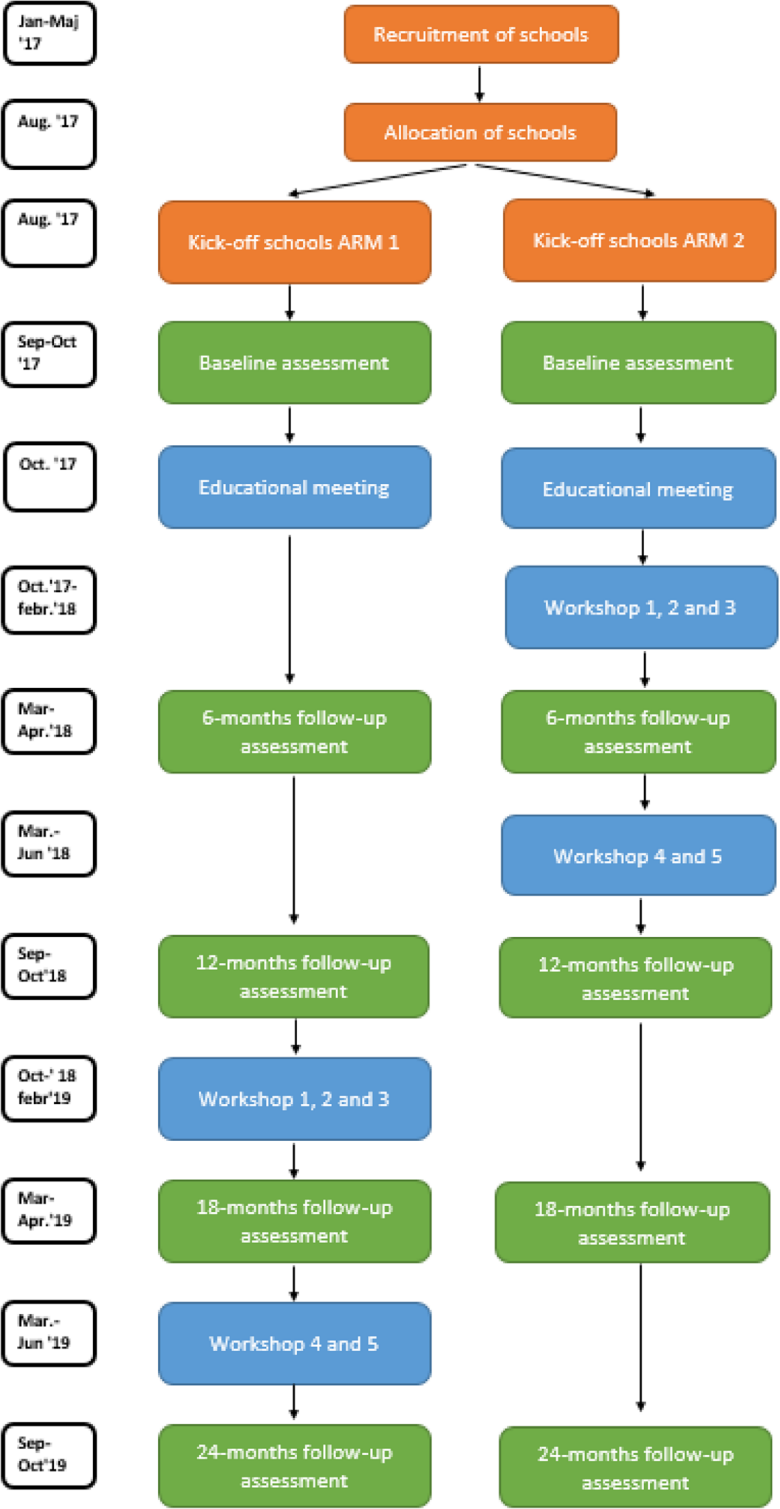
Time schedule of enrolment, implementation interventions and assessments

Figure 3 describes the time schedule of enrolment, implementation strategies and assessments.

### Sample size

The power-calculation for the proposed study is calculated on the effectiveness on employee-level (intervention effectiveness). In order to achieve 80% statistical power and a significance level of *p* < 0.05 to be able to detect an improvement in the primary outcome exhaustion with 30% among employees in the intervention group compared with employees in the control group, an intra-cluster correlation has been weighted (ICC = 0.005) and about 400 people in total are required (200 people in the intervention group and 200 people in the control group). This required the participation of at least 18 schools.

## Methods

### Assignment of interventions

#### Allocation

Primary schools are randomized to either ARM 1 or ARM 2 after stratification in blocks by municipality and school size (number of students per school). This is done by a computer-generated randomization-list. Randomization is conducted prior to the baseline measurement due to logistical reasons, as the educational meeting and workshops needed to be scheduled so that they fit with the individual planning of each school. An independent statistician, blind to the identity of the schools and not involved within the project, conducts the randomization. The project leader informs the educational board of the municipalities and the school principals by e-mail about their allocation. After assignments to implementation strategies, neither the school management or school personnel are blinded.

### Data collection, management and analysis.

#### Data collection methods.

##### Outcome measures (implementation effectiveness)

The outcome measure of implementation effectiveness is guideline adherence. Change from baseline in adherence to the recommendations of the guideline during 6-, 12-, 18- and 24-months follow-up period are measured by questionnaire. Two questionnaires are used, one directed at the school management and one directed at the school personnel. The questionnaires are developed by the research team for the purpose of this study in order to fit with the guideline’s recommendations and can be obtained upon request from the corresponding author. In order to promote participation retention and complete follow-up, the research team hands out the questionnaires in paper form in individual envelops to school personnel during the participating schools’ regular staff meetings. During these meetings, school-personnel complete the questionnaires during working hours, which they return directly to the research team on completion. A representative of the research-team is present until all questionnaires are retrieved, in order to answer questions and explain the procedure. An electronic version of the paper-questionnaire is sent to those individuals who are not present at the meeting. Three reminders are sent to those participants not responding, with 1-week interval. No outcome-data are collected from participants who discontinue. The participants do not receive a remuneration for completing the questionnaires.

The school management ((assistant) - school-principal) complete a questionnaire containing statements related to the guideline’s recommendations. Nine items (5-point Likert Scale: “fully disagree”- “fully agree”; and do not know) are used to assess adherence to recommendation 1: *Workplaces should have well-established policies related to the social and organizational risk management* (e.g. The introduction of new employees includes a briefing of the school’s working environment document). Adherence to recommendation 2: *Employers should have knowledge on the relationship between social and organizational risks and mental ill-health* is assessed with five items (5-point Likert Scale: “fully disagree”- “fully agree”; and do not know) (e.g. We have written documents stating what the school-management needs to know regarding how the work environment affects mental ill-health). Ten items are used to assess adherence to recommendation 3: *Workplaces should regularly assess their social and organizational work environment and intervene on identified social and organizational risk factors* (5-point Likert Scale: “fully disagree”- “fully agree”; and do not know) (e.g. In the last assessment of social and organisational risk factors at the workplace, the school management and employees together developed an action plan for improvement of identified social and organisational risks at the workplace). Moreover, one item (multiple-choice) assesses when the last assessment of the social and organisational work environment had taken place.

School-personnel complete a questionnaire containing similar statements related to the guideline’s recommendations as those described above. Three items are used to assess adherence to recommendation 1: *Workplaces should have well-established policies related to the social and organizational risk management* (e.g. I am familiar with the content of our work environment documents). Adherence to recommendation 2: *Employers should have knowledge on the relationship between social and organizational risks and mental ill-health* is assessed with one item. Nine items are used to assess adherence to recommendation 3: *Workplaces should regularly assess their social and organizational work environment and intervene on identified social and organizational risk factors* (e.g. In the last assessment the activities specified in the action-plan led to noticeable improvements in the social and organizational work environment). Moreover, one item (multiple-choice) assesses when the last assessment of the social and organisational work environment took place.

##### Outcome measures (intervention effectiveness)

Primary outcome

The primary outcome is exhaustion (intervention effectiveness). Change from baseline in self-reported exhaustion during 6-, 12-, 18- and 24-months follow-up is assessed by the same questionnaire as described above. We hypothesise that adherence to the recommendations of the guideline will reduce self-reported exhaustion assessed with the exhaustion scale of the Oldenburg Burnout Inventory (OLBI) [51]. Six items of the inventory are used with answering categories ranging from (1) “strongly agree” to (4) “strongly disagree”.

Secondary outcomes

The following secondary outcomes are assessed at baseline and at 6, 12, 18 and 24 months with the same questionnaire as described under implementation effectiveness directed at the school-management and school-personnel. Long-term sick-leave is assessed by register-data from the Swedish Social Insurance Agency. An overview of each secondary outcome and a description of the items used is given below.

### Demands at work

Change from baseline in self-reported demands at work during 6-, 12-, 18- and 24- months follow-up is assessed with the Copenhagen Psychosocial Questionnaire (COPSOQ) II [42]. The COPSOQ II is a well-established instrument aimed at assessing the psychosocial work environment. COPSOQ has been widely used to assess teacher’s psychosocial work environment [39]. Three different types of demands at work are assessed: quantitative demands, work pace and emotional demands. Quantitative demands (e.g. Do you get behind with your work?) are assessed with four items, work pace is assessed with three items (e.g. Do you have to work very fast?) and emotional demands (e.g. Is your work emotionally demanding) are assessed with four items. All items have 5-point response anchors ranging from (1) “always” to (5) “never/hardly ever”.

### Work organization and job contents

Eleven items of the COPSOQ II are used to assess three domains of work organisation and job contents, namely influence at work, possibilities for development and commitment to the workplace. All items have 5-point response anchors ranging from (1) “always” to (5) “never/hardly ever”*.* Change from baseline in self-reported influence at work (e.g. Do you have a say in choosing who you work with?) during 6-, 12-, 18- and 24-months follow-up is assessed with four items. Change from baseline in self-reported possibilities for development (e.g. Can you use your skills or expertise in your work?) during 6-, 12-, 18- and 24-months follow-up is assessed with four items. Change from baseline in self-reported commitment to the workplace (e.g. how often do you consider changing jobs?) during 6-, 12-, 18- and 24-months follow-up is assessed with three items.

### Interpersonal relations and leadership

Nine items of the COPSOQ II are used to assess three domains, namely social support from superior, social support from colleagues and recognition by management. Change from baseline social support from superior (e.g. How often do you talk with your superior about how well you carry out your work?) during 6-, 12-, 18- and 24- months follow-up is assessed with three items with 5-point response anchors ranging from (1) “always” to (5) “never/ never/hardly ever”. Change from baseline social support from colleagues (e.g. How often do you talk with your colleagues about how well you carry out your work?) during 6-, 12-, 18- and 24-months follow-up is assessed with three items with 5-point response anchors ranging from (1) “always” to (5) “never/hardly ever”. Change from baseline recognition by management (e.g. Is your work recognised and appreciated by the management?) during 6-, 12-, 18- and 24-months follow-up is assessed with three items with 5-point response anchors ranging from (1) “to a very large extend” to (5) “to a very small extend”.

### Presenteeism

Change in baseline presenteeism during 6-, 12-, 18- and 24-months follow-up is assessed with a single item developed and validated by Aronsson and colleagues [43]. Presenteeism is defined as coming to work despite health problems with answering categories “yes” or “no”.

### Work performance

Change in baseline work performance during 6-, 12-, 18- and 24-months follow-up is assessed by using a modified version of the *Work Productivity and Impairment Scale General Health Questionnaire* (*WPAI: GH* [44, 45]*).* The questions concern the extent to which the individual experiences that their work performance is affected by health problems and the extent to which the individual experiences that their work performance is affected by work-related problems. The response anchors range from “health problems/work-related problems have not influenced my work” (0) to “health problems/work-related problems have completely prevented me from working” (10).

### Recovery

Change in baseline recovery during 6-, 12-, 18- and 24-months follow-up is assessed with a single item [46] with 5-point response anchors ranging from (1) “never” to (5) “very often”.

### Work-life balance

Change in baseline work-life balance (e.g. my work drains so much of my energy that it has a negative effect on my private life) during 6-, 12-, 18- and 24- months follow-up is assessed with 4-items of the COPSOQ with 4-point response anchors ranging from (1) “yes, definitely” to (4) “no, not at all”.

### Work engagement

Change in baseline work engagement during 6-, 12-, 18- and 24-months follow-up is assessed with the three statements of the Utrecht Work Engagement Scale (UWES) [47] with six response anchors ranging from (0) never to (5) always, every day.

### Self-reported stress

Change in self-reported stress during 6-, 12-, 18- and 24-months follow-up is assessed with a single question [48] with five response anchors ranging from (1) “not at all” (5) “very much”. In addition to assessing change in baseline self-reported stress by questionnaire we will also assess stress by sending a validated question by text message by mobile phone with SMS-track (https://sms-track.com/) every 4th week over 12 months. This question has been used in our previous studies on stress prevention in the workplace and involves sending a Short Message Service (SMS) every week over 12 months with a high response rate and gives a good indication of changes in stress over time [49].

### Self-perceived health

Change in baseline self-perceived health during 6-, 12-, 18- and 24- months follow-up is assessed with a single question from the SF-12 Health Survey (Short-Form Health Survey [50] with 5-point response anchors ranging from (1) excellent to (5) bad.

### Sickness absence

Change in self-report baseline short-term sick leave (last 7-days) during 6-, 12-, 18- and 24-months follow-up is assessed with two items [45]. One item assesses the number of hours worked per week; the other item assesses how many days in the last 7 days the respondent has been away from work due to sickness. Change in long-term sick leave is assessed by register-data from the Swedish Social Insurance Agency. Information is collected on long-term sick leave (> 2 weeks) due to mental ill-health (F diagnoses according to ICD-10 (International Statistical Classification of Diseases and Related Health Problems). Data is collected for the entire study period (24 months) and 1 year prior to baseline. Only data on sick leave related to depression, anxiety, or stress problems (light form of mental illness) is collected. Employees provide a separate written consent for the collection of their personal sickness data from the Swedish Social Insurance Agency.

### Psychosocial safety climate

Change in baseline self-reported psychosocial safety climate (PSC) during 6-, 12-, 18- and 24-months follow-up is assessed with 12 items of the Psychosocial Safety Climate Scale [52] with 5-point response anchors ranging from (1) strongly disagree to (5) strongly agree. For example, my workplace management acts quickly to correct problems/issues that contribute to employees’ psychosocial health. The items measure the concept of psychosocial safety climate, which refers to an organizational climate for psychological health, wellbeing and safety and reflects the extent to which the senior management, according to the employees, commits to stress prevention at work [52].

#### Process evaluation

The implementation process of the educational meeting, implementation team and workshops are evaluated to investigate how well they have been implemented by collecting information on implementation outcomes as defined by Proctor and colleagues [35]. Implementation outcomes include reach, acceptability, satisfaction, feasibility, and fidelity. In addition, barriers and facilitators are assessed by interviews.

#### Reach

Reach is operationalized as the absolute number and proportion of individuals who participate in the educational meeting and workshop series. Reach is assessed through attendance lists, which are completed by the research team during the educational meetings and during each workshop.

#### Acceptability and satisfaction

Acceptability of the educational meeting and workshops is operationalized as the perception of the participants that the meeting and workshops are agreeable and satisfactory. Acceptability is assessed by questionnaire, which is completed directly after the educational meeting and after each workshop. The questionnaire includes 12 statements regarding the educational meeting/workshop structure and contents, with 5-point answering anchors ranging from (1) “to a very small extent” to (5) “to a very large extent” (e.g. The content of the meeting/workshop was relevant for implementing the recommendations of the guideline). In addition, the participants are asked to provide an overall rating for the educational meeting and workshops on a scale from 1 to 10 and to describe if any parts of the educational meeting or workshops need improving and which parts are good.

After the end of the implementation strategies, semi-structured interviews with implementation team members are performed to explore their views on the acceptability of the implementation strategies (i.e. educational meeting, implementation team and workshop series).

#### Feasibility

Feasibility is operationalized as the extent to which the implementation strategies can be successfully used and carried out within a school setting. After the end of the implementation period, semi-structured interviews with each school principal and others that have participated in the implementation strategies are performed to explore their views on the feasibility of the implementation strategies. The interview will, among others, assess the feasibility for the implementation team to meet between workshops, the feasibility of participating in the workshops and the feasibility of the frequency and duration of the educational meeting and workshops.

#### Fidelity

Fidelity is defined as the degree to which the implementation strategies are implemented as intended by the research team. Fidelity is measured by assessing 1) adherence to the protocol, 2) amount of intervention strategies delivered and 3) quality of delivery. For the educational meetings and each workshop, a time-schedule is developed. The schedule includes a fidelity column where any deviations are noted. The research-team is responsible for monitoring whether the implementation strategies are delivered according to the protocol. Procedures for monitoring adherence include making notes in the fidelity column when deviations occur for example regarding time or content. Moreover, a research-log is kept describing deviations and possible reasons for deviations.

#### Barriers and facilitators

Information on barriers and facilitators that may influence the implementation process is collected during semi-structured interviews held at 12- and 24- months follow-up with all school principals who participate in the study. An interview guide will be developed for the purpose of the study and will include questions based on CFIR (http://www.cfirwiki.net/guide/app/index.html). The framework provides a pragmatic structure for analyzing implementation factors in complex, multi-level interventions. Examples include, questions related to the school management’s perception of the advantage of implementing the guideline versus usual practice.

#### Data management

All participants receive a code and data is de-personalised. The code is kept separate from the data in a secure and locked place. Data collected during the educational meetings and workshops are first written on paper and then entered into the database by the research-team. Original paper documentation is saved and stored securely at Karolinska Institutet to allow for quality control of electronic data entry. All electronic data is stored in password protected folders on a secure data server at Karolinska Institutet to avoid unauthorized access. Measures to prevent loss of data are taken using systematic back-up routines throughout data collection and by data storage on servers with complete data back-up on a daily basis. Access to data is restricted to the research personnel working directly with data entry or analyses.

#### Statistical methods

The hypothesis is that the participants in ARM 1 when compared to the participants in ARM 2 will demonstrate improvements in exhaustion over time, as their schools will demonstrate higher adherence to the recommendations of the guideline compared to schools in the control group. Secondary hypotheses are that ARM 1 participants when compared to ARM 2 participants will demonstrate significant improvements in experience of job-demands, work organisation and job contents, interpersonal relations and leadership, presenteeism, work performance, recovery, work-life balance, work engagement, self-perceived stress, self-rated health, sickness absence and psychosocial safety climate.

Intention-to-treat analyses will be performed and where relevant, compared to per-protocol analyses. The aim of the statistical analyses is to examine the between group differences over time in adherence to the recommendations of the guideline (implementation effectiveness) and in exhaustion and the other individual level outcomes. The outcome variable adherence to the recommendations of the guideline will be operationalized into level of adherence based on the questionnaire developed by the research team. We will use mixed effects regression models to test the hypotheses of between groups difference in exhaustion. This analysis will allow for the modelling of data on school personnel, nested within the schools. Moreover, mixed models structure allows for inter-personnel and inter-schools heterogeneity through random effects such as individual intercepts and slopes over time. If potential confounders are unevenly distributed and if this is likely to affect the results when the two arms are compared, these factors will be adjusted for in the analyses. Possible interaction effects on the outcomes will be checked for. If they are statistically significant, stratified analyses will be considered. Finally, exploratory analysis will be performed to compare schools with high adherence to the recommendations of the guidelines compared to schools with low adherence. These analyses will for example examine differential effects on exhaustion and secondary outcomes. In addition, we will examine potential moderators such as school size, stability in school management etc. A detailed statistical analysis plan will be developed in collaboration with a statistician prior to starting the data analysis.

Regarding the qualitative data, interviews will be digitally recorded and transcribed verbatim. The recordings and texts will then be cross-checked for accuracy by the research team. We will employ thematic analysis with a deductive approach according to CFIR for the analysis of the interviews [53]. In the first step, the transcripts will be read through and listed to get an overall insight into the content. In the second step, the text is explored in line with the aim of identifying barriers and facilitators and coded accordingly. In the third step, codes will be collated into potential themes. In the last steps, themes will be reviewed, defined and named. Analysis will be conducted by researchers of the research team who have not been involved in the implementation strategies, e.g. educational day or workshops.

### Methods: monitoring

#### Data monitoring

The implementation strategies are aimed at improving the management of social and organisational risks at the workplace, serious risks or undesired effects for participants are not to be expected. Participants are not exposed to any excess risk because of participating in this trial. We did therefore not deem it necessary to have a data monitoring committee. However, an unintended effect of the assessment of stress and related social and organisational risk factors could be that participants expect personalized support for dealing with identified risk factors. Unintended effects will be monitored by the research-team by assessing whether participants have experienced any negative effects because of participating in the study.

### Ethics and dissemination

The Regional Ethical Board in Stockholm (dr. nr. 2017/984–31/5) has approved the proposed study. The study complies fully with current ethical requirements regarding the handling and storage of personal data and regarding the written informed consent process in accordance with Sweden’s Personal Data Act and Secrecy Act. Any amendments made to the protocol are communicated to the Regional Ethical Board in Stockholm, the International Standard Randomized Controlled Trial Register and if applicable to scientific journals, where the results of the study are published. Potential participants are given adequate information on both the possible risks and the potential benefits of their involvement in the study in order to allow them to make informed decisions about whether or not to participate. First, an oral presentation is given at each school during which the research team inform all employees about the study. Second, participants will need to provide a written informed consent. Before completing the informed consent form, potential participants receive an information letter describing the purpose of the study, research approach, voluntary participation and data collection process. The information letter also explains that participants can withdraw from participation at any time of the study. Some people could consider the information collected a breach of integrity, even though the collected information is not linked to individual’s personal identification number. Moreover, there may be concerns that the schools will have access to individual answers. The information letter therefore clearly states that data is only collected for the purpose of the study, data is only presented on group-level and no personal data will be shared with the school. The personal identification number is coded in accordance with a serial number so that these are de-identified. The code key is saved, which will enable individuals to request an extract of the collected data and demand that information on him/her to be destroyed without any given reason. One possible risk is that the participants in the focus group interviews could potentially perceive the interview questions as sensitive. This risk is dealt with by stating that participation is voluntary and that participants decide for themselves what information they wish to share. If the participant wishes, he or she can read through and comment on the interview printout. On demand of the participant, information on him/her can be destroyed without any given reason.

#### Dissemination policy

The results of the study will be disseminated through scientific papers, seminars and popular science reports. Authorship eligibility guidelines include actively contributing to scientific papers, professional writers will not be used. The results of the study will be presented to the schools and municipalities through a seminar. Moreover, information will be disseminated through stakeholder channels (e.g. the electronic newsletter of the Swedish Association of School Principals and Directors of Education). Social media (e.g. Twitter) will be used to disseminate information on the study both regarding the process and results of the study. At the end of the study period, a popular science report will be written describing the study and its results. In case of positive results, a manual describing the implementation plan will be developed.

## Discussion

This study protocol describes the design of a cluster randomized controlled trial comparing two approaches for implementing the *Guideline for the prevention of mental ill-health at the workplace* within schools. Schools that receive support in implementing the guideline through a multifaceted implementation intervention are expected to be more responsive to working in a structured and systematic manner with the management of social and organisational risks at their workplace and consequently reduce risk factors for mental ill-health, compared to schools that receive support through a single-strategy implementation intervention. This study will provide valuable knowledge on implementation and intervention effectiveness, and on the implementation process, including the identification of possible barriers and facilitators. To our knowledge, this is the first study that aims to support employers with the management of social and organisational risks at the workplace through the implementation of a guideline for the prevention of CMDs. We are aware of only one previous study that has evaluated implementation strategies aimed at the implementation of an occupational health guideline directed at mental health problems among workers. This study was focused on improving adherence to the guideline among occupational physicians and establishing faster return-to-work among sick-listed employees [54].

### Methodological considerations

Several methodological matters need to be taken into consideration. One of the study’s weaknesses is that we cannot exclude the risk of contamination between schools within the same municipality, i.e. that the group receiving only the educational meeting may receive information and/or material from the workshops. This concern is discussed with the schools and educational board of the municipalities that have received clear instructions not to discuss the content of the workshops or share any workshop documents. Another weakness is that we had to randomize schools before the baseline measurement was performed. This was necessary for the schools to plan the activities during their busy academic school year. Finally, we cannot exclude that the constantly changing school-context (e.g. high turnover of personnel and school principals, reorganizations, national and local policy changes etc.) may influence effectiveness. It is likely that schools in both arms are equally affected by the changing school-context. In order to consider these contextual changes, information is collected from the municipalities’ educational board and school principals during the entire study period.

However, the study also has several strengths, such as the structured and theory-based development of the implementation strategies. The structured approach includes the identification of barriers and facilitators, implementation strategies that target identified barriers and facilitators and the use of model (COM-B) in the development of the strategies. Moreover, two planning-workshops with, among others, school-principals were held to tailor the content of the strategies to the school-context. A second strength is the systematic approach used to plan the evaluation. A recent review on the use of theory (or models and frameworks) to plan or evaluate guideline implementation concluded that only half of the guideline implementation studies were based on theory and many provided little details about how theory was used [33]. To evaluate guideline implementation, we use Proctor and colleagues’ framework for dissemination and implementation research [35]. In addition, CFIR [21] is used to identify barriers and facilitators that may have influenced the implementation process. By doing this the study will generate new insight into the effectiveness of theory-informed implementation. Another strength of this study is that we will collect information on implementation effectiveness and intervention effectiveness (i.e. risk factors for mental ill-health), in addition to conducting a thorough process evaluation, including the identification of barriers and facilitators. This will provide valuable knowledge on whether successful implementation of the recommendations in the guideline will result in changes in the employees’ perceived social and organizational risk factors, mental ill-health and sick leave. The process evaluation will in turn help to explain the effectiveness of the chosen strategies and any contextual factors that might have influenced effectiveness.

### Impact of study results

Mental ill-health is today the leading cause of sick leave among the working population in Sweden, a professional group that is especially vulnerable are teachers. The problems related to mental ill-health are extensive for the individual due to suffering and stigmatization, and expensive for society. There is therefore an urgent need to gain more knowledge on effective strategies that prevent the occurrence of CMDs. If the multifaceted implementation intervention proves successful, schools will be able to systematically manage social and organisational risks at their workplace and reduce the risk for CMD and sick leave among its employees.

## Data Availability

Not applicable.

## Abbreviations

CFIR: Consolidated Framework for Implementation Research
CMD: Common mental disorders
COPSOQ: Copenhagen Psychosocial Questionnaire
EU-OSHA: European Agency for Safety and Health at Work
ICC: Intra-Cluster Correlation
ICD-10: International Statistical Classification of Diseases and Related Health Problems - Tenth Revision
OECD: Organization for Economic Cooperation and Development
OLBI: Oldenburg Burnout Inventory
PSC: Psychosocial Safety Climate
SF-12: Health Survey: Short-Form Health Survey
SMART-goals: Specific, Measurable, Achievable, Relevant and Time-bound goals
SMS: Short Message Service
UWES: Utrecht Work Engagement Scale
WPAI:GH: Work Productivity and Impairment Scale General Health Questionnaire
